# Dislocation of the Cervical Vertebra

**Published:** 1893-10

**Authors:** 


### DISLOCATION OF THE CERVICAL VERTEBRA.

G. L. Walton, of Boston, states (*N. Y. Medical Journal*) that this condition is not rare, though frequently overlooked. Reduction by extension, usually attempted, was unsuccessful. Spontaneous reduction had occurred, showing that direction was more important than force. This fact had led him to the discovery of the method theoretically correct, as shown by manipulation of the vertebræ, and by experiments upon the cadaver, made by Dr. Richardson, of Boston, and himself. The method had been recently demonstrated in practice by Dr. Beach, at the Massachusetts General Hospital, with successful result:

Suppose the left articular process of one vertebra has slipped forward over that of the vertebra below and fallen into the depression anterior to the articular process : This bends the head to the left and turns the face to the right. The reduction is accomplished by extending the head diagonally backward to the right, so as to elevate the articular process, after which rotation to the left replaces the displaced vertebra. The transverse processes on the right act as a fulcrum. The ligaments, which hold the vertebra firmly in the false position, make no opposition to this manœuver, which requires no force. In bilateral dislocation the same movements should be made, first on one side, reducing it to unilateral displacement, then on the other.

Dr. J. J. Putnam said that cases were more numerous than was supposed, and cited the instance of a man injured by a fall, who accidentally died through carelessness in his removal to a hospital. Dr. Sachs asked if this method would be practical in other than recent cases. Dr. Walton replied that the operation had been performed in one case of about ten days' standing. A case in which bilateral dislocation was produced spontaneously, with perfect recovery, would lead him to recommend the attempt at reduction, even after a number of months, should there be no doubt as to the diagnosis.—*Medical Standard.*

